# Supplementary material for: Study of Caspase 8 mutation in oral cancer and adjacent precancer tissues and implication in progression
Source: PLoS One. 2020 Jun 3;15(6):e0233058. doi: 10.1371/journal.pone.0233058 (PMC7269231; doi:10.1371/journal.pone.0233058)
Supplement: Attachment — Submitted filename: Response to Reviewers.docx [file pone.0233058.s002.docx]

**Editor’s note:**

1. Cover letter has been modified
2. Ethical Statement has been updated in Method and submission form
3. Participant consent has been updated in method and submission form
4. Additional information on statistical method has been updated in method
5. Participant recruitment has been elaborated in method
6. Sample size has been explained in method
7. Data availability has been mentioned and uploaded
8. “Data not shown” has been corrected

**Reviewers’ note:**

We like to thank reviewers for their excellent comments to improve the manuscript. All comments have been addressed.

Response to Reviewers

**1) The authors argue that Caspase 8 is a driver of tumorigenesis in the oral cavity. What is the evidence that this is the case. Previous studies have found that up to 34% of HNSCC carry mutations in Caspase 8. If Caspase 8 is a driver, it must be a driver in only some cancers.**

Authors’ response: Role of *CASP8* has been elucidated in lines 63-67. Since, inactivating mutations of *CASP8* gives tumor growth advantage, we do expect to see a higher frequency of mutated gene in cancer.

We have shown that *CASP8* may have worked as driver mutation in some of the leukoplakia and tumor tissues (lines 215-224).

**2) In the Discussion, the authors argue in Lines 191-194 that more mutations are necessary to drive transformation. If this is correct, is Caspase 8 really a driver. Is it necessary, sufficient, both, or neither**.

Authors’ response: In a study by Tomasetti *et al*, PNAS, 2015(PMID 25535351), the authors showed that sequential accumulation of mutations (that is, three mutations in tumor suppressors or oncogenes) are necessary to develop tumors. Along these lines we hypothesize, that mutations in *CASP8* may be an early event in some of the leukoplakias followed by mutations in other driver genes which then lead to tumorigenesis. This is further elaborated in the manuscript for clarity (lines 215-224).

**3) Have any animal models been generated to study the role of Caspase 8 in HNSCC?**

Authors’ response: We found reports on CASP8-deficient mice models of colorectal cancer but not for HNSCC. But study on cell lines with CASP8 mutation has been reported and mentioned in text (lines 65-67).

**4) Need to describe the exclusion criteria for the study.Need to explain specifically why 13 patients were dropped.**

Authors’ response: These queries have been mentioned in lines 92-94.

**5) In the Discussion, the authors state in lines 208-210 that “molecular changes are not always reflected in histopathological features”. This statement needs to be clarified or justified. Are they referring to Caspase 8 or other genes?**

Authors’ comments: The authors here refer to molecular changes such as *CASP8* mutations which may not show distinct phenotype change in histopathological features of leukoplakia and may not show direct correlation with grades of lesion severity (Lines 242-251).

**6) Dr. Liskay and his laboratory staff demonstrated that a field of Apc-deficient cells was critical to the establishment of a tumor in the colon using elegant mouse models (Carcinogenesis 35:237, 2014). In fact, they demonstrated the size of the field is critical. This finding support statement in lines 230-232 in the Discussion.**

Authors’ response: This excellent observation has been mentioned in manuscript with reference (lines 238-241).

**7) Recent studies have demonstrated that all mutant alleles in cancer drivers are not created equal, e.g., different mutations in KRAS have different degrees of oncogenic potential (Nat Commun 8: 2053, 2017). Seems that this point should be discussed because the authors present information regarding the different mutations observed in Caspase 8**

Authors’ response: This important observation has been mentioned in discussion section (lines 228-231, 366-268).

**Minor**

**1) Tables 1 and 2 could easily be combined into one.**

Authors’ response: Table 1 and 2 are combined as per reviewers’ suggestion.

**2) Table 3 does not need to restate the shared mutation in columns 3 and 4.**

Author’s response: Table is modified as per reviewers’ suggestion.

**3) The grammar needs to be corrected throughout the manuscript.**

Author’s response: We have re-written many sections of the manuscript to improvise the language.

**Reviewer #2: This study reports on the investigation if CASP8 mutations which are common in oral cancers, are also present in pre-neoplastic lesions, and as such investigate whether CASP8 mutations are an early event in the transition to oral cancer.
Does figure 1 show the mutations of the patients reported in this report? If so make this clear in the figure caption.**

Author’s response: Yes, mutations shown in Figure 1A were observed in 15 of the tumors included in this study. To compare it with mutation present in other HNSCC patients’ samples of TCGA cohorts, we have included Figure 1B. Figure 1B shows CASP8 mutations reported in a TCGA samples. Figure caption has been changed accordingly.
